# Culture‐Free Detection of Crop Pathogens at the Single‐Cell Level by Micro‐Raman Spectroscopy

**DOI:** 10.1002/advs.201700127

**Published:** 2017-07-10

**Authors:** Qinhua Gan, Xuetao Wang, Yun Wang, Zhenyu Xie, Yang Tian, Yandu Lu

**Affiliations:** ^1^ State Key Laboratory of Marine Resource Utilization in South China Sea College of Oceanology Hainan University Haikou Hainan Province 570228 China; ^2^ Inspection and Quarantine Technology Center Shandong Entry‐Exit Inspection and Quarantine Bureau Qingdao Shandong Province 266002 China; ^3^ Hisense Company Qingdao Shandong Province 266555 China; ^4^ Shanghai Hesen Biotech Co LTD Shanghai 201802 China; ^5^ Institute of Deep‐sea Science and Engineering Chinese Academy of Sciences Sanya 572000 China; ^6^ Laboratory of Tropical Biological Resources of Ministry of Education Hainan University Haikou 570228 China

**Keywords:** crop pathogens, culture‐free detection, micro‐Raman spectroscopy, single cells

## Abstract

The rapid and sensitive identification of invasive plant pathogens has important applications in biotechnology, plant quarantine, and food security. Current methods are far too time‐consuming and need a pre‐enrichment period ranging from hours to days. Here, a micro‐Raman spectroscopy‐based bioassay for culture‐free pathogen quarantine inspection at the single cell level within 40 min is presented. The application of this approach can readily and specifically detect plant pathogens *Burkholderia gladioli* pv. *alliicola* and *Erwinia chrysanthemi* that are closely related pathogenically. Furthermore, the single‐bacterium detection was able to discriminate them from a reference Raman spectral library including multiple quarantine‐relevant pathogens with broad host ranges and an array of pathogenic variants. To show the usefulness of this assay, *Burkholderia gladioli* pv. *alliicola* and *Erwinia chrysanthemi* are detected at single‐bacterium level in plant tissue lesions without pre‐enrichment. The results are confirmed by the plate‐counting method and a genetic molecular approach, which display comparable recognition ratios to the Raman spectroscopy‐based bioassay. The results represent a critical step toward the use of micro‐Raman spectroscopy in rapid and culture‐free discrimination of quarantine relevant plant pathogens.

## 1 Introduction

Global food security is being threatened by the ever‐expanding human population, which is expected to rise from 7.2 billion to 9.6 billion by 2050.^1^ The situation is among other further aggravated by the reduced crop production that results from the spread of pathogens via trade and transport. Pathogen attacks can decrease the world's annual harvest by up to 20% through yield losses, and contribute to a further 10% loss during postharvest.^2^ Human activity intensifies phytopathogen dispersal and has severe implications for native biodiversity and the health of ecosystems. Therefore, the prediction and ranking of potential invasive pathogens is pivotal in determining quarantine regulations and is also an important part of national security policies.^3^

Conventional methods for the detection and identification of plant pathogens mainly rely on recognizing specific microbiological and biochemical components.^4^ These methods depend on phenotypic features or metabolic reactions, both of which are hard to standardize and have high false positive rates.^5^ Genomic analyses (e.g., 16S rDNA) and serological assays (e.g., enzyme‐linked immunosorbent assays) have been developed to detect pathogens with relatively high accuracy.^6^, ^7^ However, the inherent disadvantage of these methods is the time‐consuming process of culture enrichment, which ranges from days for fast‐growing bacteria to weeks for slower growing species. Moreover, certain plant pathogens do not readily grow in a laboratory environment. Thus, the inspection period for some pathogens can become quite prolonged. This delayed diagnosis frequently leads to adverse effects ranging from mild symptoms to catastrophes and is jeopardizing food security.^8^ Hence, faster, ideally culture‐free techniques for pathogen identification are necessary to successfully avoid these food security threats. For this reason, recent research has focused on finding alternative approaches for pathogen identification.

Comprehensive biochemical information can be provided by noninvasive optical spectroscopy techniques, e.g., infrared, hyperspectral imaging and Raman spectroscopy.^9^, ^10^ With very low background noise of aqueous samples, Raman spectroscopy is especially well‐suited for biological applications.^9^ A single‐cell Raman spectrum of bacterium represents a sum of Raman spectra of all cell components. It provides comprehensive information about the cell (e.g., nucleic acids, proteins, carbohydrates, and lipids), and could enable the distinction of various strains at the unicellular level with appropriate chemometrical methods based on comprehensive reference databases.^10^, ^11^, ^12^ Moreover, confocal micro‐Raman spectroscopy provides high spatial resolution and allows the differentiation of bacterial cells at the micrometric scale.^13^ In particular, surface‐enhanced Raman scattering spectroscopy is able to enhance the signal by 11 orders of magnitude, which is sufficient for single‐molecule detection.^14^ Therefore, micro‐Raman spectroscopy has been utilized to monitor bacterial development, probe cellular stress response, analyze functions of microbial communities, and detect clinically relevant bacteria in laboratory settings.^11^, ^12^, ^15^, ^16^, ^17^, ^18^, ^19^, ^20^, ^21^, ^22^, ^23^, ^24^ We also utilized Raman microspectroscopy to probe bacterial metabolism and interactions^9^, ^25^ and isolate single cells.^26^ However, the potential application of this technique to quarantine relevant phytopathogens remains elusive. On the other hand, the diagnosis of plant pathogens from the plant host can be complicated by the fact that plants may be infected by multiple pathogens simultaneously or individually in nature or in a laboratory setting. Considering the largely identical morphological symptoms, the identification of different pathogens from the same plant host can be difficult.

Here we demonstrated that micro‐Raman spectroscopy can be utilized to detect and discriminate crop pathogens (i.e., *B. gladioli* pv. *alliicola* and *E. chrysanthemi* from a same host). Furthermore, this reagentless and noninvasive method was adapted to culture‐free determination of plant pathogens and demonstrated sensitivity comparable to those of genomic analysis. Our results represented a significant step in the creation of Raman‐based quarantine tools for rapid, culture‐independent, and label‐free detection of microorganisms in regulated plant samples.

## 2 Result and Discussion

### 2.1 Single Bacterial Cell Detection with a Micro‐Raman Spectroscopy

**Figure 1:**
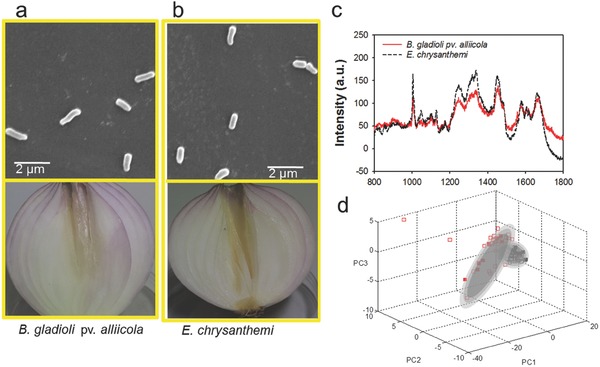
Microscopic images, pathogenic symptoms, and micro‐Raman spectra of *B. gladioli* pv. *alliicola* and *E. chrysanthemi*. a) Microscopic images of *B. gladioli* pv. *alliicola* and the pathogenic symptoms in onion tissue infected by *B. gladioli* pv. *alliicola*. b) Microscopic images of *E. chrysanthemi* and the pathogenic symptoms in onion tissue infected by *E. chrysanthemi*. c) Comparison of the *B. gladioli* pv. *alliicola* and *E. chrysanthemi* Raman spectra. d) Discrimination of the *B. gladioli* pv. *alliicola* (red squares) and *E. chrysanthemi* (black squares) Raman spectra. For each species, 30 bacterial spectra were used as training sets while ten bacterial spectra were used to validate the model. The open scatter symbols are for training spectra, and the filled symbols are for the testing spectra.

The two pathogens *B. gladioli* pv. *alliicola* and *E. chrysanthemi* were isolated from onion bulbs by plate streaking method following a standard procedure in our lab^4^, ^6^, ^27^ based on pathogen symptoms and genotyping (16S rDNA). Microscopic observation of *B. gladioli* pv. *alliicola* and *E. chrysanthemi* revealed a largely identical morphology with rod shape and similar size (**Figure** **1**a,b). By the bare eye, a separation of the different species is not possible. Symptoms of diseased onion bulbs caused by *B. gladioli* pv. *alliicola* can vary from the rotting of individual scales to a mushy rot of whole bulb (Figure 1a and Figure S1, Supporting Information). The symptoms of *E. chrysanthemi* infection morphologically resemble *B. gladioli* pv. *alliicola* infection (Figure 1b and Figure S1, Supporting Information). The visual differences of the diseased individuals were subtle, or even imperceptible, to trained personnel. Therefore, *B. gladioli* pv. *alliicola* and *E. chrysanthemi* were selected as model species to explore the ability of micro‐Raman spectroscopy to diagnose plant pathogens that are closely related pathogenically.

Experimental conditions, particularly growth stages and nutrition situations, may influence the Raman spectra of bacteria, even of the same species.^12^ To minimize any unwanted bias that could influence the Raman spectra measurements, *B. gladioli* pv. *alliicola* and *E. chrysanthemi* were grown in NB medium. Moreover, all bacterial samples were washed with distilled water immediately prior to spectrum collection to minimize background signal contributed by different culture media.^23^ Raman spectroscopy has been demonstrated to be effective in discriminating microorganisms either in synchronized cultures, the stationary growth phase of unsynchronized cultures, or random growth phases of unsynchronized cells.^28^ Further investigations revealed that the single‐cell spectra could be used to differentiate between growth phases of a single species, but these differences did not obscure the overall interspecies discrimination.^29^ On the other hand, a previous study^30^ found that the Raman spectra of cells in stationary phase were characterized by sharp, distinct signals whereas the spectra of cells from other phases, e.g., early logarithmic phase, exhibited low signal‐to‐noise (S/N) ratios with broad bands. Moreover, most plant pathogens always lie in a stationary state in natural environments.^28^ Stationary‐phase cells were thus used for Raman measurements. To increase the variability of the data set, several batches (three biological replicates per species) were included. Within each replicate, the spectrum was collected for no less than 60 cells at a randomly selected position to evade any potential variations caused by different cell architectures.^30^

Although *B. gladioli* pv. *alliicola* and *E. chrysanthemi* display pathogenic and morphological similarities, they have distinctive dominant spectra. Despite the largely similar signal patterns, the Raman signals of *E. chrysanthemi* show obvious increases in the bands 1004 cm^−1^ (assigned to phenylalanine), 1260 cm^−1^ (alkyl = C‐H *cis* stretches), 1340 cm^−1^ (carbohydrate C‐H2 deformation and C‐O‐H bending), and 1445 cm^−1^ (CH_2_ deformation vibrations) (Figure 1c). Moreover, the spectra of *B. gladioli* pv. *alliicola* and *E. chrysanthemi* were analyzed by using principal component analysis (PCA). The background noise originating from the instruments, optical elements, and surrounding environments can complicate the interpretation of Raman spectra. Thus, the initial Raman spectra of plant pathogens underwent quality control with Labspec 5 and our recently developed signal processing approach (rDisc),^31^ which has been demonstrated to be effective in noise eliminating, wavelet transform denoising, baseline correction, and signal normalization. These processed, high‐quality spectra were then classified using PCA analysis as described by Xie.^28^ PCA used a series of linear combinations of the original Raman features to capture the inherent variability present in samples and maximize the Raman variance. As for the scatter plot of the Raman spectra of each species, a 3D subspace was obtained. Thirty bacterial spectra were randomly selected as training sets while ten spectra were utilized to validate the correctness of the PCA model.^28^ The 90% confidence interval for each of the clusters was represented by the outermost gray ellipsoids. *B. gladioli* pv. *alliicola* and *E. chrysanthemi* could be readily distinguished, with the application of PCA analysis (Figure 1d). The partial overlap is due to the orientation of the 3D graph.

### 2.2 Discriminate *B. gladioli* pv. *alliicola* and *E. chrysanthemi* from Typical Quarantine‐Relevant Plant Pathogens

The diagnosis of *B. gladioli* pv. *alliicola* and *E. chrysanthemi* symptoms on onions can be complicated by the presence of other pathogens. To test the sensitivity of micro‐Raman detection for *B. gladioli* pv. *alliicola* or *E. chrysanthemi*, we constructed a reference Raman spectral library for typical quarantine‐relevant plant pathogens with a relatively deep phylogenetic diversity (Figure S1, Supporting Information). To include a broad range of characteristics of pathogens, five pathogen characteristics were considered for the library (five genera, two species from a same genus, two subspecies from a same species, different pathovar strains and a wide range of hosts; please see detailed description in the Supporting Information text 1).

**Figure 2:**
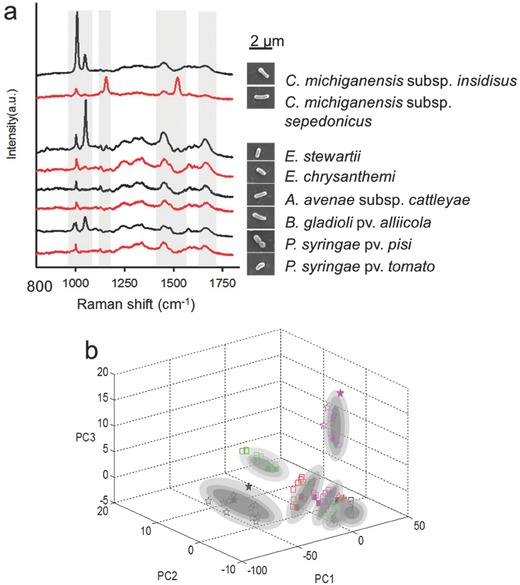
Discriminate *B. gladioli* pv. *alliicola* and *E. chrysanthemi* from typical quarantine‐relevant plant pathogens. a) Microscopic images and average Raman spectra of eight different pathogenic strains. The positions of the Raman bands at 1004, 1048, 1155, 1445, 1519, and 1655 cm^−1^ are highlighted. Scale bar represents 2 µm. b) Discrimination of the Raman spectra of the eight typical plant pathogens. The pathogens are represented as follows: *A. avenae* subsp. *cattleyae* (green pentagons), *B. gladioli* pv. *alliicola* (black squares), *C. michiganensis* subsp. *insidiosus* (green squares), *C. michiganensis* subsp. *sepedonicus* (pink pentagons), *E. chrysanthemi* (pink squares), *E. stewartii* (red squares), *P. syringae* pv. *pisi* (black pentagons), and *P. syringae* pv. *tomato* (red pentagons). The open scatter symbols are for training spectra, and the filled symbols are for the testing spectra.

**Figure** **2**a demonstrates the average Raman spectra of the selected plant pathogens, along with their scanning electron microscopic images. These pathogens resemble to each other in shape (i.e., rod‐shaped), but have difference in size, yet without statistical significance. Variation of Raman spectra for 40 bacteria cells of each strain was shown in Figure S2 (Supporting Information). Despite the distinctive dominant spectra, spectral patterns of some pathogens are nearly impossible to differentiate (e.g., *E. stewartii* and *E. chrysanthemi*; *A. avenae* subsp. *cattleyae* and *B. gladioli* pv. *alliicola*). Thus, a chemometric data processing method is required for a reliable analysis. The spectra were analyzed using a PCA model to further evaluate the performance of the Raman‐based diagnosis for plant pathogens on a larger scale. Recognition rate for Raman spectra of each pathogen was shown in **Table** **1**. Due to the viewing angle, an overlap appears when 40 cells are included for each strain. Thus, in Figure 2b, for each pathogen, ten bacterial spectra were used as training sets and two spectra were used as testing sets as described by Xie et al.^28^ The eight quarantine relevant pathogens were clustered into eight distinct groups (Figure 2b). All of the testing spectra were correctly located indicating an effective discrimination of these pathogens by micro‐Raman spectroscopy. Although the *A. avenae* subsp. *cattleyae*, *B. gladioli* pv. *alliicola*, and *P. syringae* pv. *tomato* clusters appeared to overlap, they could be separated when the 3D plot was rotated along one of its axes. Taken together, *B. gladioli* pv. *alliicola* and *E. chrysanthemi* can be discriminated from the reference Raman spectral library of typical quarantine‐relevant plant pathogens. Moreover, these selected plant pathogens can be separated by micro‐Raman spectroscopy with the application of PCA analysis.

**Table 1:** Recognition rate for Raman spectra of various plant pathogens. Thirty bacterial spectra were randomly selected as training sets while ten spectra were utilized to validate the correctness of the PCA model

Strain	Training set no.	Validation set no.	Wrong classified no.	Recognition rate
*Acidovorax avenae* subsp. *cattleyae*	30	10	1	90%
*Burkholderia gladioli* pv. *alliicola*	30	10	2	80%
*Clavibacter michiganensis* subsp. *insidisus*	30	10	0	100%
*Clavibacter michiganensis* subsp. *sepedonicus*	30	10	0	100%
*Erwinia chrysanthemi*	30	10	0	100%
*Erwinia stewartii*	30	10	0	100%
*Pseudomonas syringae* pv. *pisi*	30	10	0	100%
*Pseudomonas syringae* pv. *tomato*	30	10	0	100%

### 2.3 Culture‐Free Determination of Plant Pathogens Using Raman Fingerprints

The development of culture‐free diagnostic methods for invasive plant pathogens remains a challenge in plant quarantine. Therefore, we investigated the applications of Raman spectroscopic analysis for pathogen detection in infected plant tissue. By blasting the above constructed reference Raman spectral library with spectroscopy of pathogens from infected plant tissues, we also aimed to evaluate our testing system.

**Figure 3:**
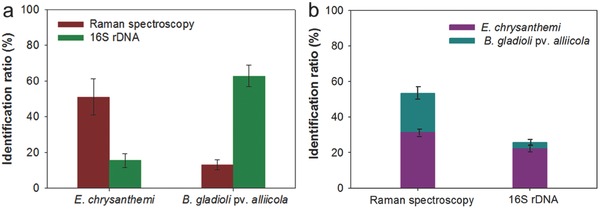
Culture‐free detection of *E. chrysanthemi* and *B. gladioli* pv. *alliicola* in onion tissues. a) Comparison of the identification ratios of micro‐Raman spectroscopy‐ and molecular marker (i.e., 16S rDNA)‐based diagnostic approaches for pathogen detection in onion infected by either *E. chrysanthemi* or *B. gladioli* pv. *alliicola*. b) Comparison of the identification ratios of micro‐Raman spectroscopy‐ or molecular marker (i.e., 16S rDNA)‐based diagnostic approaches for pathogen detection in onion infected by both *E. chrysanthemi* and *B. gladioli* pv. *alliicola*.

Healthy onion bulbs were inoculated with *B. gladioli* pv. *alliicola* and *E. chrysanthemi* either individually or simultaneously. Three biological replicates were set for each treatment. Raman measurements were taken for 15 cells from each replicate of plant tissue infected with either *B. gladioli* pv. *alliicola* or *E. chrysanthemi*. The spectra were analyzed following the procedure described in Methods. In the *E. chrysanthemi* infected replicates, 7.7 ± 1.5 cells displayed positive signals within 15 tested cells (**Figure** **3**a). An average identification ratio of 15.6 ± 3.8% was obtained for samples inoculated with *B. gladioli* pv. *alliicola* (Figure 3a). Samples that had been simultaneously inoculated with both *E. chrysanthemi* and *B. gladioli* pv. *alliicola* underwent Raman measurements for 20 cells, with a pair‐wise Euclidean distance calculation for each of the three replicates. The identification ratios for *E. chrysanthemi* and *B. gladioli* pv. *alliicola* were 31.7% and 21.7%, respectively, and the total ratio was 53.4% (Figure 3b).

The remainder of each treatment's aliquot was plated onto NA agar, with three replicates. Bacterial clones were then picked at random and identified by a 16S rDNA sequence comparison. According to the 16S rDNA sequence comparison, among the chosen 68 clones of the samples infected by *E. chrysanthemi*, polymerase chain reaction (PCR) amplification of the 16S rDNA gene was not obtained for almost 80% clones (52 clones). Nine clones were identified as *E. chrysanthemi* (13.1%), while the remaining bacteria were identified as *Pseudomonas* sp. (*n* = 3), *Enterobacter* sp. (*n* = 2), *Escherichia* sp. (*n* = 1), and *Pantoea* sp. (*n* = 1) (Figure 3a). In the samples infected by *B. gladioli* pv. *alliicola*, 23 of the 37 picked clones were identified as *B. gladioli* pv. *alliicola* (62.9%), and two were identified as *Pseudomonas* sp. (Figure 3a). No PCR amplification was obtained for the remaining 12 clones. In the samples co‐inoculated with *B. gladioli* pv. *alliicola* and *E. chrysanthemi*, only 22 of the 62 picked clones (35.5%) were identified by 16S rDNA. Two clones were identified as *B. gladioli* pv. *alliicola* and 14 as *E. chrysanthemi*, which translates to recognition ratios of 3.2% and 22.6%, respectively (Figure 3b). The remaining clones were identified as *Pseudomonas* sp. (*n* = 4), *Enterobacter* sp. (*n* = 1), and *Escherichia* sp. (*n* = 1). The clones identified as *B. gladioli* pv. *alliicola* or *E. chrysanthemi* by 16S rDNA gene also underwent micro‐Raman spectroscopy for pathogen identification. Ten cells were randomly selected for each sample and a high identification ratio was revealed (70–84%).

We should carefully interpret the recognition ratio of Raman spectroscopic analysis for pathogens grown in real plant samples. Compared with the reference database, the bacterial composition and culture conditions of plant samples are unknown. For example, the composition of the plant tissues (matrix) and the dwell time in tissues (“culture” time) are unknown.^12^ Furthermore, no standardized procedures can be followed during single‐cell measurements. Despite that we collected spectra of pathogens of different batches, only spectra of pathogens in the stationary growth phase were included, which limited variation in the reference database. Inclusion of as much as possible variations into the classification reference data set is necessary to cope with these challenges.

As for the 16S rDNA detection method, it should be noted that the number of clones found in NA agar may not reflect the relative abundance of active cells in the sample. When the well‐described biases of utilizing enrichment medium are taken into account, a compromise should be considered for the identification ratio reflected by 16S rDNA sequence comparison. On the other hand, several environmentally ubiquitous taxa (*Pseudomonas* sp., *Enterobacter* sp., *Escherichia* sp., and *Pantoea* sp.) were identified from the NA agar through 16S rDNA sequencing comparison. Additionally, the 16S rDNA of a considerable amount of clones (62.3%) was not amplified through PCR using experimentally validated robust primers. These observations may allow the speculation that the current practice for manipulating isolated plant cankers (plant cankers were surface sterilized using 75% ethanol prior to pathogen detection) was susceptible to contamination. This may also result in, to some degree, the negative signals of Raman spectroscopy assay. An expanded reference spectroscopy database will allow a more systematic evaluation. Taken together, micro‐Raman spectroscopy is a rapid and noninvasive method for the culture‐free detection of plant pathogens that demonstrates an identification ratio that is comparable to the commonly used molecular method (i.e., 16S rDNA sequence comparison).

## 3 Conclusions

We have developed a fast and sensitive method that uses micro‐Raman spectroscopy to identify quarantine relevant plant pathogens at the single‐cell level without the need for enrichment. By using micro‐Raman spectroscopy, plant pathogens can be detected quickly and accurately in real plant tissues. As the first step, a reference database was established. Then real plant samples was tested to evaluate applicability of the reference model for culture‐free detection of *B. gladioli* pv. *alliicola* and *E. chrysanthemi* in onion cankers. This bioassay is rapid (≈40 min from plant sample collection to detection and analysis, which could be further shortened), convenient, and highly selective, with a sensitivity comparable to genetic methods (for which pre‐enrichment is necessary), demonstrates its promising applications in *in situ* detection of plant pathogens, particularly for detection of uncultured pathogens.

Restrictively, it has to be mentioned that the herein presented proof‐of principle is only done with the most important regulated plant pathogens. To further ensure the sensitivity to discriminate each pathogen, a standard practice should be developed and the number of species within the same genus, subspecies within the same species and pathovars, as well as the number of isolates per strain should be increased (Figure S3, Supporting Information). Moreover, the development of *in situ* single‐cell sequencing technology^32^, ^33^ can be utilized to further examine and improve the accuracy of Raman microspectroscopy for crop pathogen detection. As the standardizing of the operating procedures (sampling methodologies, spectrum collection, signal processing and analysis) and the expansion of scope and depth of reference spectroscopy database, a great promising is held for the practical utility of micro‐Raman spectroscopy in plant quarantine.

## 4 Experimental Section

*Plant Pathogens and Single‐Bacterium Sample Preparation*: The plant pathogens used in this study were introduced in Supporting Information text 1 and listed in Table S1 (Supporting Information). Strains were preserved at −80 °C in the laboratory. Prior to Raman measurement, all pathogens were inoculated, activated, and cultivated in NB medium (10 g L^−1^ peptone, 3 g L^−1^ beef extract, 5 g L^−1^ NaCl, pH 7.0). All strains were cultured at 28 °C with 150 rpm shaking. Bacterial cells were collected and washed three times with axenic water. The precipitate was resuspended at ≈10^5^ colony‐forming units per mL (CFU mL^−1^). Aliquots of 10 µL, containing ≈10^3^ cells, were transferred to a CaF_2_ slides. To facilitate an easy capture of a single cell, the prepared slides were dried on a clean bench. Control samples were obtained by using the same experimental procedures, but without the addition of bacteria. Three samples were prepared for each test and analyzed simultaneously to obtain statistically reliable results. All strains were cultured and manipulated in a Biosafety Level III Laboratory.

*Microscopy and Micro‐Raman Spectroscopy*: Raman spectra were captured using a laser with an excitation wavelength of 532 nm (inVia Reflex, Renishaw, England). Raman spectra of each cell were acquired with a 100 × magnifying dry objective with laser power being ≈5 mW on the sample (while the power at the laser was 50 mW). The diameter of the spot was 1 µm. An acquisition time of 10 s was applied to all the pathogens. The spectral range was recorded from 593 to 2133 cm^−1^, with a spectral resolution of 2 cm^−1^ achieved by a 600 groove mm^−1^ grating in the spectrograph. This region included all the Raman bands necessary for the identification of bacteria. Intrasample repeatability was evaluated and mean spectra for each cell were obtained. For each strain, bacteria were collected and sampled for scanning electron microscopy (SEM) analysis.

*Spectral Analysis of Reference Raman Spectra Library*: Raman spectra of the same species could differ based on both the accuracy of the experimental device and electronic noise. Therefore, it was essential to summarize the intraclass distribution pattern. The raw spectra were preprocessed (baseline‐correction and normalization) using LabSpec 5 software (HORIBA Scientific, Orsay, France). Spectra with an extremely poor S/N ratio or unusually large background fluorescence were discarded. The remaining spectra were subjected to quality control with the recently developed signal processing approach (rDisc)^30^ followed by PCA. The data processing was carried out with Matlab R2010a (The MathWorks, Inc., Natick, MA, USA).

Both peak intensity and peak‐position information were included in the similarity evaluation to increase its reliability. Pearson's correlation was selected as the method for evaluating the intensity of the peaks. The leave‐one‐out method was used for the first validation of the PCA analysis models.^28^ The spectra of all but one of the reference strains were used to generate the respective models.^34^ The resulting prediction models were then tested by identifying the strain that was left out based on its spectrum. Each strain was handled with such a procedure.

*Culture‐Free Sample Preparation and Spectral Analysis*: Inocula were prepared by growing *B. gladioli* pv. *alliicola* and *E. chrysanthemi* on NB plates at 28 °C for 24 h. Bacterial cells were collected and suspended (10^7^–10^8^ CFU mL^−1^) in sterile water. The bacterial suspension was inoculating into the healthy onion bulbs through a wound. In negative controls, sterile water was used instead of a pathogen solution. Three parallels were established for each treatment. Following inoculation, the onion bulbs were placed in an incubator under conditions of thermal insulation and moisture retention. The susceptible tissue was collected for Raman measurement after 48 h of incubation.

The inoculated samples were ground with a blade into a paste‐like consistency. Each ground onion sample was added to 2 mL of axenic water and mixed with a vortex for 1 min. The entire sample was then filtered through sterile gauze, after which it was washed with 1 mL axenic water and centrifuged (5000 rpm, 3 min) three times. The supernatant was removed and the samples were re‐suspended in 100 µL of axenic water. Each sample was then divided into two aliquots; one aliquot was used for micro‐Raman spectroscopy, and the other one was assayed with the conventional plating method.

Dissimilarity was computed using 30 randomly chosen Raman spectra from the reference Raman spectra database for each pathogen. The first step of culture‐free spectral analysis was the calculation of a value for each spectrum based on the pair‐wise Euclidean distance between the analyzed spectrum and each of the 30 reference spectra. In supervised identification, the pair‐wise Euclidean distance has been calculated to generate a Normal distribution model for each species in which μ is the average Euclidean distance value in the training database and σ is the variance of all spectra (Formula 1). A Normal distribution model was created for each of the eight species

(1) $$f\left(x\right)=\frac{1}{\sigma \sqrt{2\pi }}e^{-\frac{{\left(x-\mu \right)}^{2}}{2\sigma^{2}}}$$

Eight values were obtained for each culture‐free spectrum and these values were then compared with μ of corresponding Normal distribution models. The closer the obtained value was to μ, the more probable it was that the culture‐free spectra were from that strain. To minimize the potential bias from background interference and the spectroscopic similarity of different species, the two most similar reference strains were set as candidate positives for each culture‐free spectrum. Finally, given that plant pathogens normally infect certain host(s), the candidate strains were further probed according to available knowledge about their host range to evaluate whether they could infect the investigated plant sample. If the candidate strain could infect such a plant, it was regarded as a positive strain.

*DNA Isolation, 16S rDNA Amplification, and Phylogenetic Analysis*: Single clones were picked from NA plates and grown overnight in liquid NA medium at 28 °C with 150 rpm shaking. Genomic DNA was isolated and analyzed following our laboratory's previously described procedure.^27^ The 16S fragments were amplified with gene‐specific primers, 16SF (5′‐AGAGTTTGATCATGGCTCAG‐3′) and 16SR (5′‐ACGGTTACCTTGTTACGACTT‐3′). PCR fragments of the expected length were purified and sequenced. Parsed hits for all species were aligned using CLUSTALW (European Molecular Biology Laboratory, Cambridgeshire, UK).^35^ Gaps and ambiguously aligned sites were removed using gBlock.^36^ Sequences that caused either aberrant alignments or for which the real identity could not be confirmed were removed manually. Phylogenetic analyses were performed in MEGA 5.0 using a maximum likelihood method.^37^ Bootstrap support values were estimated using 100 pseudo‐replicates.

## Conflict of Interest

The authors declare no conflict of interest.

## Supporting information

Supplementary

## References

[1] Population Division of the Department of Economic and Social Affairs of the United Nations Secretariat, https://www.un.org/development/desa/en/news/population/un‐report‐world‐population‐projected‐to‐reach‐9‐6‐billion‐by‐2050.html (accessed: March 2017).

[2] E. C. Oerke , H. W. Dehne , Crop Prot. 2004, 23, 275.

[3] D. R. Paini , S. P. Worner , D. C. Cook , P. J. De Barro , M. B. Thomas , Nat. Commun. 2010, 1, 115. 21081913 10.1038/ncomms1118

[4] Q. Gan , Y. Li , X. Shao , Y. Wang , Acta Phytophysiol. Sin. 2011, 38, 183.

[5] Y. He , G. P. Munkvold , Plant Pathol. 2012, 61, 837.

[6] Q. Gan , Y. Wang , X. Wei , X. Shao , C. Liu , Y. Li , Acta Phytophysiol. Sin. 2014, 4, 346.

[7] M. F. Clark , Annu. Rev. Phytopathol. 2003, 19, 83.

[8] R. N. Strange , P. R. Scott , Annu. Rev. Phytopathol. 2005, 43, 83. 16078878 10.1146/annurev.phyto.43.113004.133839

[9] Y. Wang , W. E. Huang , L. Cui , M. Wagner , Curr. Opin. Biotechnol. 2016, 41, 34. 27149160 10.1016/j.copbio.2016.04.018

[10] P. Kubryk , J. S. Kolschbach , S. Marozava , T. Lueders , R. U. Meckenstock , R. Niessner , N. P. Ivleva , Anal. Chem. 2015, 87, 6622. 26010835 10.1021/acs.analchem.5b00673

[11] S. Kloß , P. Rösch , W. Pfister , M. Kiehntopf , J. Popp , Anal. Chem. 2015, 87, 937. 25517827 10.1021/ac503373r

[12] S. Kloß , B. Kampe , S. Sachse , P. Rösch , E. Straube , W. Pfister , M. Kiehntopf , J. Popp , Anal. Chem. 2013, 85, 9610. 24010860 10.1021/ac401806f

[13] R. M. Jarvis , R. Goodacre , Chem. Soc. Rev. 2008, 37, 931. 18443678 10.1039/b705973f

[14] E. J. Blackie , E. C. L. Ru , P. G. Etchegoin , J. Am. Chem. Soc. 2009, 131, 14466. 19807188 10.1021/ja905319w

[15] D. Berry , E. Mader , T. K. Lee , D. Woebken , Y. Wang , D. Zhu , M. Palatinszky , A. Schintlmeister , M. Schmid , B. Hanson , Proc. Natl. Acad. Sci. USA 2015, 112, E194. 25550518 10.1073/pnas.1420406112 PMC4299247

[16] T. Liu , K. Tsai , H. Wang , Y. Chen , Y. Chen , Y. Chao , H. Chang , C. Lin , J. Wang , Y. Wang , Nat. Commun. 2011, 2, 538. 22086338 10.1038/ncomms1546

[17] S. Feng , J. Pan , Y. Wu , D. Lin , Y. Chen , G. Xi , J. Lin , R. Chen , Sci. China: Life Sci. 2011, 54, 828. 21809036 10.1007/s11427-011-4212-8

[18] K. Maquelin , L. P. Choo‐Smith , H. P. Endtz , H. A. Bruining , G. J. Puppels , J. Clin. Microbiol. 2002, 40, 594. 11825976 10.1128/JCM.40.2.594-600.2002 PMC153356

[19] K. Maquelin , L. P. Choo‐Smith , T. van Vreeswijk , H. P. Endtz , B. Smith , R. Bennett , H. A. Bruining , G. J. Puppels , Anal. Chem. 2000, 72, 12. 10655628 10.1021/ac991011h

[20] A. J. Berger , Q. Zhu , J. Mod. Opt. 2003, 50, 2375.

[21] A. Assaf , C. B. Y. Cordella , G. Thouand , Anal. Bioanal. Chem. 2014, 406, 4899. 24908409 10.1007/s00216-014-7909-2

[22] V. Velusamy , K. Arshak , O. Korostynska , K. Oliwa , C. C. Adley , Biotechnol. Adv. 2010, 28, 232. 20006978 10.1016/j.biotechadv.2009.12.004

[23] L. Teng , X. Wang , X. Wang , H. Gou , L. Ren , T. Wang , Y. Wang , Y. Ji , W. E. Huang , J. Xu , Sci. Rep. 2016, 6, 34359. 27756907 10.1038/srep34359 PMC5069462

[24] S. Pahlow , S. Stöckel , S. Pollok , D. Cialla‐May , P. Rösch , K. Weber , J. Popp , Anal. Chem. 2016, 88, 1570. 26705822 10.1021/acs.analchem.5b02829

[25] Y. Wang , Y. Song , Y. Tao , H. Muhamadali , R. Goodacre , N. Zhou , G. M. Preston , J. Xu , W. E. Huang , Anal. Chem. 2016, 88, 9443. 27588325 10.1021/acs.analchem.6b01602

[26] Y. Wang , Y. Ji , E. S. Wharfe , R. S. Meadows , P. March , R. Goodacre , J. Xu , W. E. Huang , Anal. Chem. 2013, 85, 10697. 24083399 10.1021/ac403107p

[27] Q. Gan , Y. Lu , X. Shao , T. Sun , Y. Li , C. Liu , J. Phytopathol. 2014, 162, 190.

[28] C. Xie , J. Mace , M. A. Dinno , Y. Q. Li , W. Tang , R. J. Newton , P. J. Gemperline , Anal. Chem. 2005, 77, 4390. 16013851 10.1021/ac0504971

[29] W. Huang , R. Griffiths , I. Thompson , M. Bailey , A. Whiteley , Anal. Chem. 2004, 77, 4452. 10.1021/ac049753k 15283587

[30] P. Rösch , M. Harz , M. Schmitt , K.‐D. Peschke , O. Ronneberger , H. Burkhardt , H.‐W. Motzkus , M. Lankers , S. Hofer , H. Thiele , J. Popp , Appl. Environ. Microbiol. 2005, 71, 1626. 15746368 10.1128/AEM.71.3.1626-1637.2005 PMC1065155

[31] S. Sun , X. Wang , X. Gao , L. Ren , X. Su , D. Bu , K. Ning , BMC Bioinformatics 2015, 16, S15. 26681607 10.1186/1471-2105-16-S18-S15 PMC4682421

[32] C. Trapnell , Genome Res. 2015, 25, 1491. 26430159 10.1101/gr.190595.115 PMC4579334

[33] T. Nawy , Nat. Methods 2014, 11, 29. 10.1038/nmeth.2777 24524137

[34] D. H. Kim , R. M. Jarvis , J. W. Allwood , G. Batman , R. E. Moore , E. Marsden‐Edwards , L. Hampson , I. N. Hampson , R. Goodacre , Anal. Bioanal. Chem. 2010, 398, 3051. 20957472 10.1007/s00216-010-4283-6

[35] R. Chenna , H. Sugawara , T. Koike , R. Lopez , T. J. Gibson , D. G. Higgins , J. D. Thompson , Nucleic Acids Res. 2003, 31, 3497. 12824352 10.1093/nar/gkg500 PMC168907

[36] G. Talavera , J. Castresana , Syst. Biol. 2007, 56, 564. 17654362 10.1080/10635150701472164

[37] K. Tamura , G. Stecher , D. Peterson , A. Filipski , S. Kumar , Mol. Biol. Evol. 2013, 30, 2725. 24132122 10.1093/molbev/mst197 PMC3840312

