# Supplementary material for: Culture‐Free Detection of Crop Pathogens at the Single‐Cell Level by Micro‐Raman Spectroscopy
Source: Adv Sci (Weinh). 2017 Jul 10;4(11):1700127. doi: 10.1002/advs.201700127 (PMC5700641; doi:10.1002/advs.201700127)
Supplement: Supplementary file 1 — Supplementary [file ADVS-4-na-s001.pdf]

Copyright WILEY-VCH Verlag GmbH & Co. KGaA, 69469 Weinheim, Germany, 2017.

ADVANCED SCIENCE
Open Access

Supporting Information

for *Adv. Sci.,* DOI: 10.1002/advs.201700127

Culture-Free Detection of Crop Pathogens at the Single-Cell Level by Micro-Raman Spectroscopy

*Qinhua Gan, Xuetao Wang, Yun Wang, Zhenyu Xie, Yang Tian, and Yandu Lu**

Supporting Information

Culture-free detection of crop pathogens at the single-cell level by micro-Raman spectroscopy

Qinhua Gan, Xuetao Wang, Yun Wang, Zhenyu Xie, Yang Tian, Yandu Lu[*]

SUPPLEMENTARY TEXT 1

Selection of plant pathogens for Raman spectroscopy

The most common regulated phytopathogens with deep phylogenetic diversity were selected. For instance, among the genus *Erwinia*, *E. stewartii* (also known as *Pantoea stewartii* subsp. *stewartii*) is a causal agent of Stewart's wilt of susceptible corn cultivars[2] while *E. chrysanthemi* causes both soft rot and wilt diseases in a variety of agriculturally important plants, including ornamental plants and food crops. Therefore, both *E. stewartii* and *E. chrysanthemi* represent the main pathogen transmission route in the international trade[9] and are considers as an A2 quarantine pest. On the other hand, *Clavibacter michiganensis* causes serious bacterial diseases in a broad range of plant species. Based on genetic markers, host specificity, and bacteriological characteristics, five *C. michiganensis* subspecies have been identified, of which three (*C. michiganensis* subsp. *michiganensis*, *C. michiganensis* subsp. *sepedonicus* and *C. michiganensis* subsp. *insidiosus*) are classified as quarantine organisms worldwide, making them subject to international quarantine regulations. *C. michiganensis* subsp. *michiganensis* causes bacterial wilt and canker of tomato (*Solanum lycopersicum*)[4] while *C. michiganensis* subsp. *insidiosus* is a seed-borne

pathogen that causes wilting and stunting in alfalfa, which is the most important forage crop worldwide.[2] *P. syringae* is a gram-negative, rod-shaped bacterium with polar flagella. Over 60 different *P. syringae* pathovars have been discovered over an extremely wide host range.[7] *P. syringae* pv. *pisi* is a seedborne pathogen that causes pea bacterial blight while *P. syringae* pv. *tomato* causes the bacterial speck of tomato. Both strains are distributed worldwide and can initiate spells of potentially devastating disease that will severely reduce yield and seed quality.[6] *Burkholderia gladioli* is a gram-negative, rod-shaped bacteria that causes disease in both humans and plants.[19] In plants, onion is the most common host of *B. gladioli* pv. *alliicola*. Plants may be infected by multiple pathogen strains, simultaneously or individually, that demonstrate very similar, or even identical, pathogenic symptoms. For instance, we isolated two pathogens *B. gladioli* pv. *alliicola* and *E. chrysanthemi* from onion bulbs. The visual differences of the diseased individuals were subtle, or even imperceptible, to trained personnel (**Figure** 1). Thus, both *B. gladioli* pv. *alliicola* and *E. chrysanthemi* were included in this study to explore the ability of micro-Raman spectroscopy to diagnose plant pathogens that are closely related pathogenically.

Table S1. Plant pathogens used in this study. Abbreviation: EPPO: European and Mediterranean Plant Protection Organization.

Pathogen Strains	Sources	EPPO code	EPPO rank	Hosts
Acidovorax avenae subsp. *cattleyae*	Institute of Plant Protection, Chinese Academy of Agricultural Sciences, State Key Laboratory for Biology of Plant Diseases and Insect	ACVRAC	NA[I]	*Orchid* [1,4,3]

	Pests			
Burkholderia gladioli pv. *alliicola*	Laboratory of Microbiology Gent Bacteria Collection (BCCM/LMG)	PSDMGA	NA	*Allium cepa*, *Tulipa gesneriana, Narcissus tazeta* [3]
Clavibacter michiganensis subsp. *insidisus*	China General Microbiological Culture Collection Center (CGMCC)	CORBIN	A2	*Medicago sativa*, *Medicago falcate, Medicago* spp., *Lotus corniculatus*, *Melilotus alba*, *Onobrychis viciifolia* and *Trifolium* sp. [3]
Clavibacter michiganensis subsp. *michiganensis*	CGMCC	CORBMI	A2	*Lycopersicon esculentum*, *Solanaceae* spp.
Erwinia chrysanthemi	Our lab	ERWICH	A2 (EU[II])	*Apium graveolens*, *Allium sativum*, *Allium* spp., *Solanum tuberosum*, *Arctium lappa*, *Philodendron* sp., *Phalaenopsis* spp.*, Oncidium* spp., *Dendrobium* spp., *Paphiopedilum* spp.
Erwinia stewartii	American Type Culture Collection	ERWIST	A2	*Zea mays*, *Euchlaena mexicana*, *Tripsacum dactyloides*, *Coxi lachrymajobi*, *Euchlaena perennis*, *Setaria lutescens*, *Sorghum vulgare*, *Sorghum vulgare*, *Setaria italica*, *Panicum miliaceum*, *Avena satival*, *Schlerachne punctata* [1]
Pseudomonas syringae pv. *pisi*	BCCM/LMG	PSDMPI	A1	*Pisum sativum*, *Dolichos lablab*, *Lathyrus odoratus*, *Vicia sepium* [2]
Pseudomonas syringae pv. *tomato*	BCCM/LMG	PSDMTM	NA	*Lycopersicon esculentum*, *Capsicum annuum*, *Solanum melongena*, *Solanum nigrum* [2]

Note: The EPPO code and rank have been queried in the web-based EPPO Global Database, which aims to gather all pest-specific information (http://www.eppo.int/). Although some parts of the database are still under development (e.g. the ranks for *Acidovorax avenae* subsp. *cattleyae*, *Burkholderia gladioli* pv. *alliicola*, and *Pseudomonas syringae* pv. *tomato*), it currently contains scientific, common names,

and ranks for the majority of plant pathogens. I, Not Available; II, *Erwinia chrysanthemi* is categorized as A2 in European.

References

[1] M. Feng, D. Z. Kong, W. B. Wang, L. Q. Liu, S. S. Song, C. L. Xu, *Sensors* **2015**, *15*, 4291.
[2] H. Kajiwara, *J. Microbiol. Methods* **2016**, *120*, 1.
[3] P. M. Michener, J. K. Pataky, D. G. White, *Plant Dis.* **2002**, *86*, 1031.
[4] F. Zhang, M. Zou, Y. Chen, J. Li, Y. Wang, X. Qi, Q. Xue, *Biosens. Bioelectro.* **2014**, *51*, 29.
[5] A. J. Gonzalez, E. Trapiello, *Int. J. Syst. Evol. Micr.* **2014**, *64*, 1752.
[6] J. Yasuhara-Bell, G. Marrero, A. M. Alvarez, *Eur. J. Plant Pathol.* **2014**, *140*, 655.
[7] H. Ali, K. Cholam, *Res. J. Chem. Environ.* **2011**, *15*, 486.
[8] A. Martín-Sanz, M. P. de la Vega, J. Murillo, C. Caminero, *Phytopathology* **2013**, *103*, 673.
[9] A. Martin-Sanz, M. P. de la Vega, J. Murillo, C. Caminero, *Plant Pathol.* **2012**, *61*, 1063.
[10] G. E. Stovold, J. Bradley, P. C. Fahy, *Australas. Plant Path.* **2001**, *30*, 73.
[11] D. Cun, W. Zhao, Y. Du, *Plant Quarantine* **2007**, *21*, 210.
[12] M. Scortichini, D. D'Ascenzo, M. Rossi, *J.Plant Pathol.* **2005**, *87*, 244.
[13] C. J. Lee, J. T. Lee, J. H. Kwor, B. C. Kim, W. Park, *Australas. Plant Path.* **2005**, *34*, 287.
[14] European and Mediterranean Plant Protection Organization. *EPPO Bulletin* **2010**, *40*, 353.
[15] European and Mediterranean Plant Protection Organization. *EPPO Bulletin* **2006**, *36*, 111.
[16] European and Mediterranean Plant Protection Organization. *EPPO Bulletin* **2016**, *17*, 291.
[17] A. T. Saad, H. M. A. Hassan, *EPPO Bulletin* **2000**, *30*, 341.

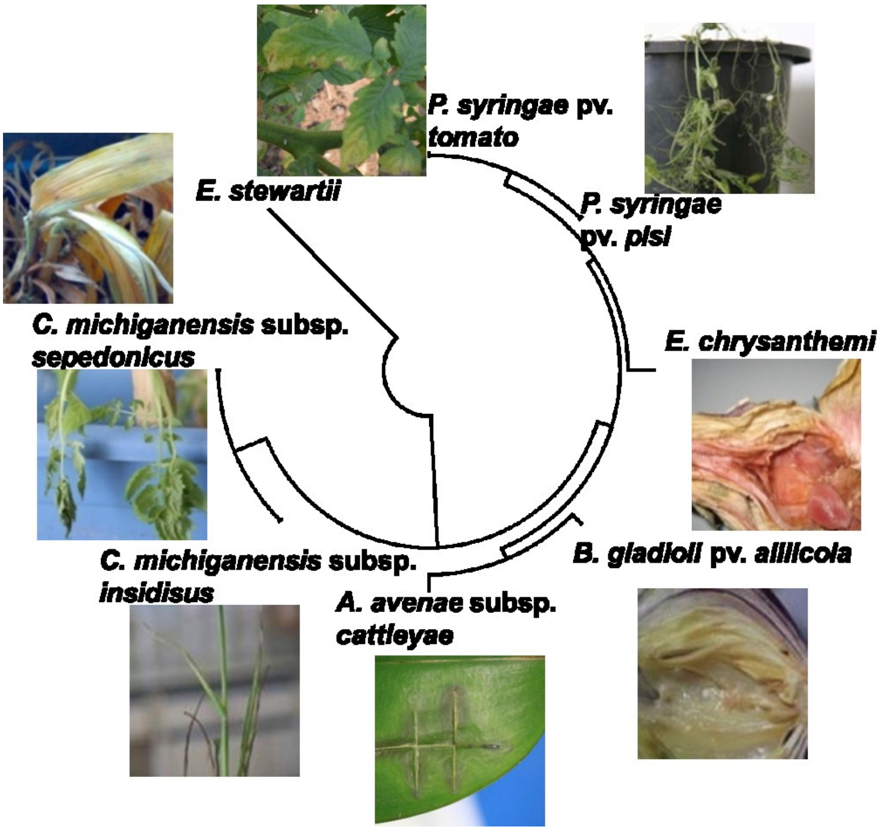

Figure S1. Pathogenic symptoms and phylogenetic relationships of the plant pathogens included in this study. The phylogenetic tree is based on the results from distance matrix analyses of 16S rDNA sequences.

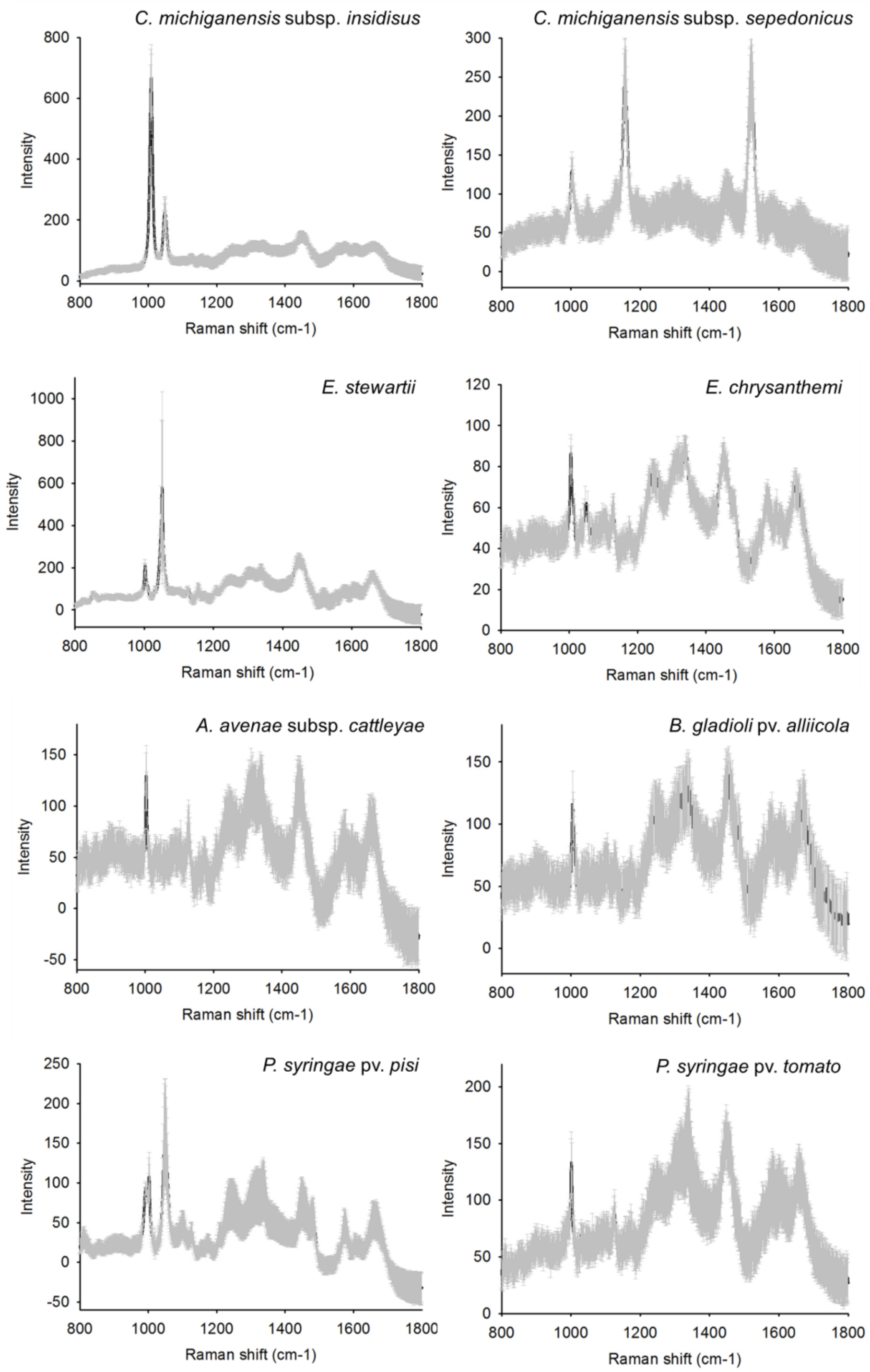

Figure S2. Comparison of varitation of single-cell Raman spectra. Varitation of Raman spectra of bacteria cells for 40 continuous measurements. Standard derivation is shown in gray.

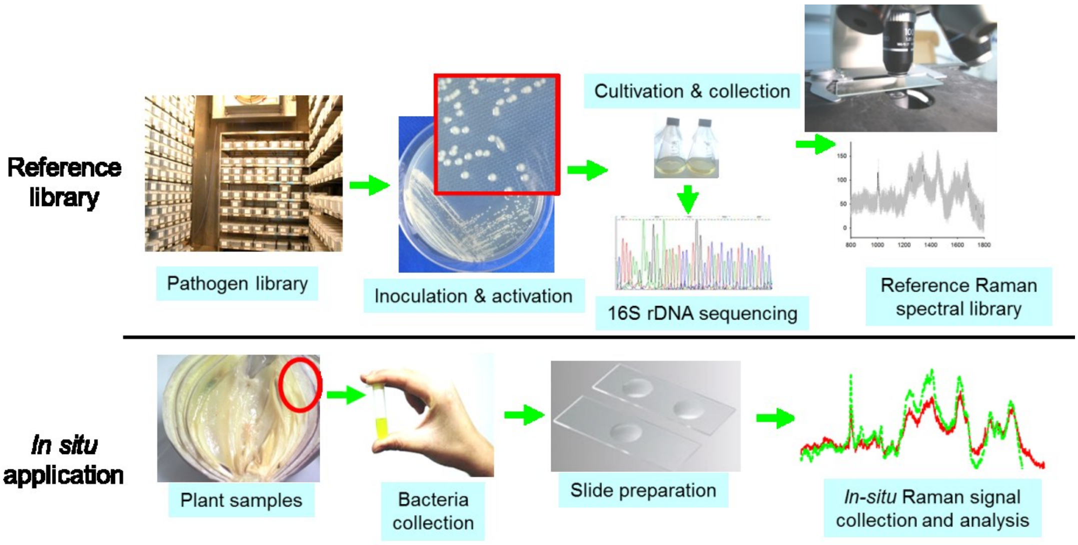

Figure S3. Streamlined operating procedures for the detection of plant pathogens using micro-Raman spectroscopy. A wider range of pathogens at a different taxonomic levels are inoculated and pure-cultivated. Raman spectral analysis and 16S rDNA sequence comparison are simultaneously performed for each strain to establish a reference Raman spectral library. Next, the susceptible plant tissues are collected and culture-free samples are prepared for Raman measurement. The obtained culture-free spectra are used to search in the Raman spectral library for candidate positives. Combined with available knowledge about host range of certain pathogen, positive strain should be identified. Meanwhile, the culture-free spectra of this strain will be included in the spectral library to expand the spectral coverage for each strain. If no positive strain has been found in the library, this strain would be taken as an unknown and cultivated in proper condition for 16S rDNA sequence identification. Spectra of this unknown should also be included in the library. Finally, Raman spectra are collected from a wider range of pathogens (genera, species, subspecies, pathovar,

and strains); moreover, for each pathogen, spectra of a larger number of isolates at conditions of both culture-free and pure-culture are included.
